# Supplementary material for: Accuracy and social motivations shape judgements of (mis)information
Source: Nat Hum Behav. 2023 Mar 6;7(6):892–903. doi: 10.1038/s41562-023-01540-w (PMC10289897; doi:10.1038/s41562-023-01540-w)
Supplement: Supplementary file 1 — Supplementary Sections 1–12. [file 41562_2023_1540_MOESM1_ESM.pdf]

nature human behaviour

Article

https://doi.org/10.1038/s41562-023-01540-w

Accuracy and social motivations shape judgements of (mis)information

In the format provided by the authors and unedited

Table of Contents

S1: Extended Results 2

S2: Manipulation Texts 8

S3: Headline-Level Analysis 15

S4: Bayes Factors for Integrative Data Analysis 17

S5: Mean Effects Sizes for All Participants and Separately for Republicans and Democrats 18

S6: Full Regression Models for Integrative Data Analysis 19

S7: Full Relative Importance Analysis for Integrative Data Analysis 20

S8: Example Stimuli 22

S9: Question Wording 23

S10: Results for Continuously Coded Outcome Variables 27

S11: Study 3 Results Including Additional News Items 28

S12: Signal Detection Analysis 29

Supplementary References 30

S1: Extended Results

Study 1

Analysis of Type of Headlines Impacted. To explore what kind of headlines the incentives impacted specifically, we conducted a 2 (incentives vs. control condition) X 2 (true headlines vs. false headlines) X 2 (politically-congruent versus politically-incongruent) mixed-design ANOVA with the percentage of articles rated as accurate as the dependent variable. There was a main effect of condition, $F(1, 460) = 12.71, p < 0.001, \eta^2_G = 0.01$, political congruence, $F(1, 460) = 263.50$, $p < 0.001$, $\eta^2_G = 0.11$, and veracity of the headlines, $F(1, 460) = 5.58, p < 0.001$, $\eta^2_G = 0.27$. There was also an interaction effect between the incentives and political congruence of the headlines, $F(1, 460) = 8.00, p = 0.01, \eta^2_G = 0.004$, and between the incentives and the veracity of the headlines $F(1, 460) = 7.77$, $p = 0.003$, $\eta^2_G = 0.004$.

Following up on these interaction effects with Tukey HSD post-hoc tests, we found that the incentives primarily increased belief in politically-incongruent true news ($M = 51.53$, 95% CI = [47.36, 55.70]) when compared to the control condition ($M = 38.25$, 95% CI = [34.41, 42.08]), $p < 0.001$, $d = 0.43$. When controlling for multiple comparisons with Tukey HSD post-hoc tests, incentives had no effect on politically-incongruent false news ($p = 0.444$), politically-congruent false news ($p = 0.999$), or politically-congruent true news ($p = 0.472$). In other words, the effect of the incentives was driven by an increase in belief in news from the opposing party.

Study 2

Analysis of Type of Headlines Impacted. To test what types of headlines were affected by the incentives, we ran a 2 (accuracy incentive vs. no incentive) X 2 (social incentive vs. no incentive) X 2 (politically congruent vs. politically incongruent) X 2 (true headlines vs. false headlines) mixed-design ANOVA with the percent of articles rated as accurate as the dependent variable. There was a significant main effect of the accuracy incentives, $F(1, 994) = 23.44, p < 0.001, \eta^2_G = 0.01$, veracity, $F(1, 994) = 550.43, p < 0.001, \eta^2_G = 0.20$, and political congruence, $F(1, 994) = 8.99, p = 0.003, \eta^2_G = 0.002$. Furthermore, there was a significant interaction between accuracy incentives and political congruence, $F(1,994) = 8.99$, $p = 0.003$, $\eta^2_G = 0.00$, between accuracy incentives and veracity, $F(1, 994) = 29.06$, $p < 0.001$, $\eta^2_G = 0.01$, and between social incentives and veracity, $F(1, 994) = 7.613$, $p = 0.006$, $\eta^2_G = 0.00$. All other *ps* > 0.085.

Tukey HSD post-hoc tests found that there was a significant difference in the amount of *incongruent true* articles rated as accurate between the accuracy incentives condition ($M = 55.61\%$, 95% CI = [51.68, 59.54]) and the control condition (M = 37.65%, 95% CI = [33.83, 41.46]), $p < 0.001$, $d = 0.58$. However, the mixed incentives condition (M = 46.07%, 95% CI = [42.04, 51.10]) did not differ from the control condition, $p = 0.092$, once again supporting the idea that social incentives distract from accuracy incentives. The incentives once again did not impact congruent true news, incongruent false news, or congruent false news (ps > 0.148).

Analysis of Sharing Behavior. To test how incentives influenced sharing intentions, we ran another 2X2X2X2 mixed ANOVA on sharing intentions. Here, there was no effect of accuracy incentives, $F(1, 994) = 0.05, p = 0.830, \eta^2 = 0.00$, but there was a significant main effect of social

incentives, $F(1, 994) = 10.07$, $p = 0.002$, $\eta^2 = 0.00$, political congruence, $F(1, 994) = 368.96$, $p < 0.001$, $\eta^2 = 0.05$, and veracity, $F(1, 994) = 173.71$, $p < 0.001$, $\eta^2 = 0.01$. Furthermore, there was a significant interaction between social incentives and political congruence, $F(1, 994) = 20.41$, $p < 0.001$, $\eta^2 = 0.00$.

Following up on the interaction between the social incentives and political congruence, we found that those in the social incentives condition shared more politically congruent news (either true or false) ($M = 1.98$, 95% CI = [1.90, 2.05]) as compared to the control condition ($M = 1.80$, 95% CI = [1.74, 1.87]), $p = 0.015$, $d = 0.21$. Additionally, those in the mixed condition ($M = 2.02$, 95% CI = [1.94, 2.10]) shared more politically congruent news (true or false) as compared to the control condition, $p < 0.001$, $d = 0.26$. Thus, thinking about whether an article will be liked by one's party, whether or not one is incentivized to be accurate, appears to indiscriminately increase sharing of both true and false news that appeal to one's political party.

Study 3

Accuracy incentives significantly improved truth discernment, $F(1, 917) = 4.44$, $p = 0.040$, $\eta^2_G = 0.01$. Additionally, there was a significant impact of source cues on truth discernment such that source cues improved accuracy, $F(1, 917) = 8.88$, $p = 0.003$, $\eta^2_G = 0.01$. However, there was no significant interaction between the accuracy incentive condition and source cues ($p = 0.284$). Post-hoc tests revealed that truth discernment was not significantly higher in the accuracy incentive (with sources) condition ($M = 2.76$, 95% CI = [2.44, 3.09]) compared to the control (with sources) condition ($M = 2.30$, 95% CI = [2.00, 2.58]), $p = 0.110$, $d = 0.20$. Without sources, truth discernment was not significantly higher in the accuracy incentives condition ($M = 2.15$, 95% CI = [1.86, 2.45]) compared to the control condition (without sources) ($M = 2.00$, 95% CI = [1.73, 2.26]), p = 0.888, $d = 0.07$.

Also replicating Experiments 1 and 2, accuracy incentives reduced partisan bias, $F(1, 917) = 18.21$, $p < 0.001$, $\eta^2_G = 0.02$. Source cues did not impact partisan bias ($p = 0.931$), and there was no interaction between source cues and partisan bias ($p = 0.923$). Post-hoc tests found that partisan bias was 33% lower in the accuracy incentives condition (with sources) ($M = 1.17$, 95% CI = [0.90, 1.45]) compared to the control condition (with sources) ($M = 1.75$, 95% CI = [1.48, 2.01]), $p = 0.017$, $d = 0.29$. Interestingly, without sources present beside the headlines, partisan bias was also lower in the accuracy incentives condition ($M = 1.15$, 95% CI = [0.87, 1.42] compared to the control condition ($M = 1.75$, 95% CI = [1.48, 2.01]), $p = 0.012$, $d = 0.27$.

Analysis of Type of Headlines Impacted. We ran a 2 (accuracy incentive vs. no incentive) X 2 (social incentive vs. no incentive) X 2 (politically congruent vs. politically incongruent) X 2 (true headlines vs. false headlines) mixed-design ANOVA. Here, we saw a significant main effect of political congruence on perceived accuracy, $F(1, 917) = 457.79$, $p < 0.001$, $\eta^2 = 0.09$. There was also a significant main effect of veracity on perceived accuracy, $F(1, 917) = 945.35$, $p < 0.01$, $\eta^2 = 0.20$. As in Study 2, there was a significant interaction between the accuracy incentives condition and political congruence, $F(1, 917) = 18.22$, $p < 0.001$, $\eta^2 = 0.00$, a significant interaction between veracity and political congruence, $F(1, 917) = 5.65$, $p = 0.018$, $\eta^2 = 0.00$, and, importantly, a significant interaction between the accuracy incentives and source cues, $F(1, 917) = 4.71$, $p = 0.030$, $\eta^2 = 0.00$, and a significant interaction between the accuracy incentives and the veracity of the headline, $F(1, 917) = 4.71$, $p = 0.036$, $\eta^2 = 0.00$.

We then followed up on these interactions with Tukey post-hoc tests. Replicating the results of studies 1 and 2, there was a large difference in the percentage of *incongruent true* headlines rated as accurate in the accuracy incentive (with sources) condition ($M = 51.20$, 95% CI = [47.28, 55.12]) versus the control (with sources) condition ($M = 39.47$, 95% CI = [35.69, 43.34]), $p < 0.001$, $d = 0.39$. However, without sources present beside the headlines, there was no difference in the percentage of incongruent true headlines rated as accurate when comparing the accuracy incentive and control condition, $p = 0.605$, $d = 0.20$. No other post-hoc tests were significant (*ps* > 0.864). These results replicate the finding that the effects are driven by an increase in belief in politically-incongruent headlines (at least when source cues are present on the headlines).

Like in Experiment 2, there was once again no significant impact of accuracy incentives on sharing discernment ($p = 0.906$). However, there was an effect of the source cues on sharing discernment such that source cues improved sharing discernment, $F(1, 917) = 4.92$, $p = 0.027$, $\eta^2 = 0.01$. There was also no interaction between accuracy incentives and source cues on sharing discernment ($p = 0.124$).

Bayesian Analysis for Null Interactions. We then conducted Bayesian analyses for the null interactions using the “BayesFactor” package in R. This analysis used a noninformative Jeffreys prior on the variance of the normal population, and a Cauchy prior on the standardized effect size. The Bayes factor for the interaction between accuracy incentives and source cues on the amount of incongruent true news rated as true was 0.27 (±4.37%), the Bayes factor for this interaction on partisan bias was 0.10 (±3.76%), and the Bayes factor for this interaction on truth discernment was 0.18 (±5.51%). Bayes factors in this range indicate weak (or “anecdotal”) to moderate levels of evidence for the null hypothesis. Thus, this study does not provide strong evidence for null interaction effects, but also does not provide strong evidence for moderation effects.

Study 4

There was a main effect of the experimental condition on truth discernment, $F(980, 2) = 6.44$, $p = 0.002$, $\eta^2_G = 0.01$. The non-financial accuracy incentive condition did not significantly increase truth discernment (M = 2.39, 95% CI = [2.11, 2.66]) as compared to the control condition ($M = 2.07$, 95% CI = [1.82, 2.33]), $p = 0.22$, $d = 0.13$, though the financial incentive condition ($M = 2.75$, 95% CI = [2.48, 3.01]) did improve truth discernment as compared to the control condition, $p = 0.001$, $d = 0.28$, replicating studies 1-3. In other words, the non-financial accuracy motivation condition appeared to have 47% as large of an effect on truth discernment, though this effect was not significant. There was also a main effect of the condition on partisan bias, $F(980, 2) = 5.36$, $p = 0.011$, $\eta^2_G = 0.011$. The non-financial accuracy motivation condition also did not significantly decrease partisan bias ($M = 1.51$, 95% CI = [1.29, 1.73]) as compared to the control condition ($M = 1.75$, 95% CI = [1.52, 1.97]), $p = 0.31$, $d = 0.12$. However, the financial incentive condition ($M = 1.22$, 95% CI = [0.98, 1.45]) did significantly decrease partisan bias as compared to the control condition, $p = 0.003$, $d = 0.25$, replicating studies 1-3. The non-financial accuracy incentive had approximately 45% as large of an effect on partisan bias, though this effect was not significant.

Integrative Data Analysis

Analysis of Types of Headlines Impacted: We ran a 2 (accuracy incentive vs. no incentive) X 2 (social incentive vs. no incentive) X 2 (politically congruent vs. politically incongruent) X 2 (true headlines vs. false headlines) mixed-design ANOVA. There was a main effect of condition on the percentage of articles rated as accurate, $F(1, 2090) = 49.41$, $p < 0.001$, $\eta^2_G = 0.01$, as well as a significant main effect of political congruence, $F(1, 2090) = 1096.05$, $p < 0.001$, $\eta^2_G = 0.10$. There were also significant interactions between the experimental condition and political congruence, $F(1, 2090) = 34.67$, $p < 0.001$, $\eta^2_G = 0.00$, the experimental condition and veracity, $F(1, 2090) = 42.701 = 42.70$, $p < 0.001$, $\eta^2_G = 0.00$, political congruence and veracity, $F(1, 2090) = 5.62$, $p = 0.018$, $\eta^2_G = 0.00$, and an interaction between condition, political congruence, and veracity, $F(1, 2090) = 8.65$, $p < 0.001$, $\eta^2_G = 0.00$.

When adjusting for multiple comparisons using Tukey post-hoc tests, incentives had a large effect on the percentage of politically-incongruent true articles rated as accurate (M_{exp} = 51.68, 95% CI = [49.77, 53.59] vs. M_{con} = 37.39, 95% CI = [35.58, 39.21]), $p < .001$, $d = 0.47$.

Incentives had a smaller positive effect on the percentage of politically-congruent true news rated as accurate (M_{exp} = 66.93, 95% CI = [65.19, 68.67]) vs. M_{con} = 61.86, 95% CI = [60.08, 63.63]), $p = 0.001$, $d = 0.17$.

Incentives did not significantly affect belief in politically-incongruent false news (M_{exp} = 15.54, 95% CI = [14.14, 16.93]) vs. M_{con} = 12.35, 95% CI = [11.07, 13.62]), $p = 0.163$, $d = 0.13$.

Incentives also did not significant affect belief in politically-congruent false news (M_{exp} = 31.26, 95% CI = [29.45, 33.08] vs. M_{con} = 32.26, 95% CI = [30.40, 34.13]), $p = 0.993$, $d = -0.04$

Reaction Time Data. People spent slightly more time on each headline in the accuracy incentives condition (M = 16.34, 95% CI = [15.49, 17.19]) as compared to the control condition (M = 14.43, 95% CI = [13.07, 15.79]), $t(818.53) = 2.34$, $p = 0.019$, $d = 0.16$.

Partisan Differences. Conservatives showed more partisan bias than liberals: partisan bias was 2.55 points for unincentivized conservatives and 1.16 points for unincentivized liberals – a 1.40 point difference, 95% CI = [1.15, 1.64], $t(906.25) = 11.23$, $p < .001$, $d = 0.70$. Yet, this difference became 0.65 points when conservatives were incentivized to be accurate, 95% CI = [0.40, 0.90], $t(834.30) = 5.02$, $p = 0.001$, $d = 0.32$. In other words, while conservatives initially expressed more partisan bias, incentives for accuracy closed this gap in partisan bias by 53.57%.

Finally, liberals judged 1.78 (out of 4) true news headlines from the opposing party to be accurate, whereas conservatives judged 1.14 (out of 4) true news headlines from the opposing party to be accurate – a 0.65-point difference, 95% CI [0.50, 0.79], $t(1026.59) = 9.00$, p < .001, $d = 0.55$. But, when conservatives were incentivized to be accurate, they correctly identified 1.83 (out of 4) true news headlines from the opposing party to be accurate, making the difference between conservatives and liberals non-significant, *difference* = 0.05, 95% CI [-0.10, 0.20], t(918.14) = 0.63, $p = 0.528$, $d = 0.04$

There was also a gap in sharing discernment between liberals and conservatives. Sharing discernment was 0.14 for unincentivized liberals, and 0.32 for unincentivized conservatives – a gap of 0.18 points, 95% CI [0.11, 0.25], $p < .001$, $d = 0.32$. However, this gap became non-significant when conservatives were incentivized to be accurate, *difference* = 0.06, 95% CI [0.02, 0.14], $p = 0.115$, $d = 0.10$.

Addressing Multiple Interpretations. One alternate interpretation of our results is that participants were simply guessing what fact-checkers would say is true in the accuracy incentives condition rather than expressing their genuine beliefs about accuracy. However, in studies 2-4, we asked participants in the accuracy incentives condition whether they answered in a way that did not reflect their true beliefs just to receive the payment, and told participants that their answer to this question would not affect their final payment (See *S7* for question wording). Only 2.61% of participants said "yes" to this question, indicating that people reported responding in a way that reflected their true beliefs.

Another interpretation is that accuracy incentives inhibit motivated responding (also known as "expressive responding" or "partisan cheerleading") (Peterson & Iyengar, 2021; Schaffner & Luks, 2018), or partisans' tendency to purposely give incorrect answers just to express support for their own party. To address this interpretation, in study 2, we asked participants who were not in the accuracy incentives condition whether they ever said an article was true (or false) not because they actually believed it was true (or false), but because they liked (or disliked it). Only 4.52% of participants admitted to engaging in this kind of motivated responding, indicating that most participants reported answering in line with their genuine beliefs.

We also directly asked participants whether they believed their responses were influenced by the treatment conditions at the end of the experiment. In total, 18.01% of participants said they believed their judgements were influenced by the accuracy incentives, and 24.40% of participants said they believed their judgements were influenced by the task of identifying politically-congruent articles. Thus, while some participants were aware that the experimental conditions impacted how they responded, the majority did not admit to being aware of this. Furthermore, only 5.41% of participants said they would knowingly share fake news on social media.

Alternate Measures of Partisan Bias. Throughout this article, we follow past research (Batailler et al., 2021; Gawronski, 2021) by defining partisan bias as belief in politically congruent news minus belief in politically-incongruent news. Other work has defined partisan bias as the difference in truth discernment between politically congruent and politically-incongruent news (Pennycook & Rand, 2018, 2021b), which may have led to different conclusions about how much partisanship plays a role in shaping belief in true and false news headlines. However, a recent article argued that measuring partisan bias in terms of truth discernment is a "problematic conception of partisan bias," as it obscures the effects of partisanship on belief, and that "partisan bias should be understood in terms of the effect of ideology congruence on overall belief, not truth discernment" (Gawronski, 2021). Following the recommendations of this article, we also define partisan bias as the effect of political congruence on belief as opposed to defining it in terms of truth discernment. Using this definition of partisan bias, we found partisan bias to be large in our integrative data analysis (d = 0.80 for unincentivized participants, and d = 0.60 for incentivized participants), which is similar to the effect sizes found in past work (Batailler et al., 2021), and also found that incentives had a consistent impact on partisan bias across studies.

However, we also re-analyzed our data to examine the difference in truth discernment between politically-congruent and politically-incongruent headlines. Replicating prior work (Pennycook & Rand, 2021b), truth discernment was slightly higher for politically-congruent headlines as compared to politically-incongruent headlines when people were not incentivized to

be accurate, $t(1066) = 3.81$, $p < 0.001$, $d = 0.12$. When incentivized to be accurate, there was no difference in truth discernment between politically-congruent and politically-incongruent headlines, $t(1024) = -0.40$, $p = 0.691$, $d = -0.01$. Additionally, incentives significantly decreased the difference in truth discernment between politically-congruent and politically-incongruent headlines, $t(2085.13) = -2.94$, $p = 0.003$, $d = 0.13$. In other words, while people are generally better at distinguishing between truth and falsehoods from their own side as opposed to the other side, incentives led people to become just as good at discerning between truth and falsehood from their side and the other side. Thus, even with this alternate conceptualization of partisan bias, incentives had a significant effect on partisan bias. Altogether, while there may be some disagreement among researchers about how to define terms such partisan bias, with some arguing that partisan bias is an improper term as partisan differences in belief do not necessarily reflect "bias" (Pennycook & Rand, 2021a), these disagreements do not change our main findings."

S2: Manipulation Texts

Accuracy Incentives Manipulation Text

You will be presented with a series of real and fake news headlines. There are 16 headlines in total.

We are interested in your opinion about the following:

1) How accurate is the headline?
2) How likely would you be to share the headline on social media?

You will be given 60 seconds to answer these two questions about each headline.

Note: You will receive a **BONUS PAYMENT** of up to **$1.00** (£0.75) based on how many **CORRECT** answers you provide regarding the accuracy of the articles. Correct answers are based on the expert evaluations of non-partisan fact-checkers.

More specifically, if you answer 15 or more of the out 16 questions correctly, you will receive the full bonus payment of $1.00. If you answer 13 or more of the 16 questions correctly, you will receive a partial bonus payment of $0.50. Your bonus payment will be delivered to your Prolific ID. It may take a few weeks to calculate your scores and for you to receive your bonus payment.

We ask you about accuracy on a 6-point scale ranging from "extremely inaccurate" to "extremely accurate." For the purpose of this study, if the headline describes a true event, either "slightly accurate," "moderately accurate," or "extremely accurate" constitute correct responses. Similarly, if the headline describes a false event, either "extremely inaccurate," "moderately inaccurate," or "slightly inaccurate" constitute "correct" responses.

Your answers to all other questions will not contribute to your bonus payment.

After seeing each headline, questions in this condition appeared as follows:

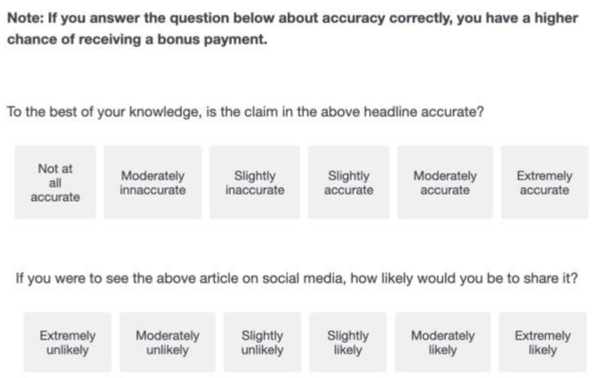

Control Text

You will be presented with a series of real and fake news headlines. There are 16 headlines in total.

We are interested in your opinion about the following:

1) How accurate is the headline?
2) How likely would you be to share the headline on social media?

You will be given 60 seconds to answer these two questions about each headline.

After seeing each headline, questions in this condition appeared as follows:

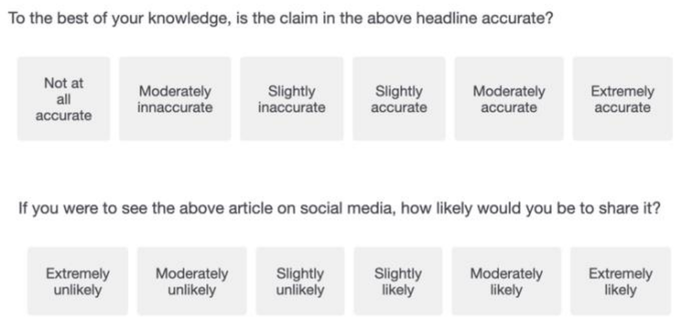

Partisan Sharing Manipulation Text

You will be presented with a series of real and fake news headlines. There are 16 headlines in total.

We are primarily interested in your opinion about the following:

1) How likely is this article to appeal to [Democrats/Republicans]?

We want to see how well you can identify articles that appeal to [Democrats/Republicans]? You will receive a **BONUS PAYMENT** of up to $1.00 (£0.75) based on **how well you identify articles that are likely to appeal to [Democrats/Republicans]?**

More specifically, we have pre-tested these articles to see how much they are liked by [Democrats/Republicans]. We want to see how close your answers are to the answers we identified in the pre-test. If you correctly identify 15 or more out of 16 articles that are liked by [Democrats/Republicans], you will receive the full bonus payment of $1.00. If you correctly identify 13 or more of the 16 articles that are liked by [Democrats/Republicans], you will receive a partial bonus payment of $0.50.

We will also ask you:

2) How accurate is the headline?
3) How likely would you be to share the headline on social media?

But, we will not give you a bonus payment based on your response to these questions. Your answers to all other questions in the survey will not contribute to your bonus payment.

The images may take a second to load. Please wait for the images to load before answering the questions.

After seeing each headline, questions in this condition appeared as follows:

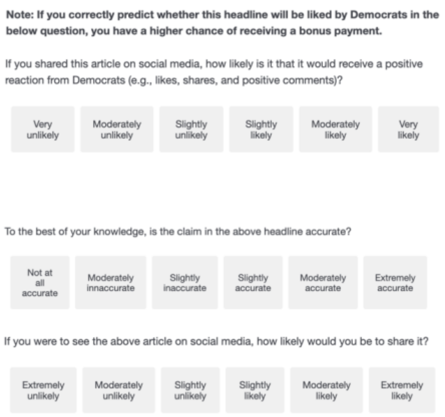

Mixed Incentives Manipulation Text

You will be presented with a series of real and fake news headlines. There are 16 headlines in total.
We are primarily interested in your opinion about the following:

1) How likely is this article to appeal to [Democrats/Republicans]?
2) How accurate is the headline?

We want to see how well you can identify articles that appeal to [Democrats/Republicans]? You will receive a BONUS PAYMENT of up to $1.00 (£**0.75**) based on ***how well you identify articles that are likely to appeal to*** [Democrats/Republicans]?

You will receive an ADDITIONAL BONUS PAYMENT of up to $1.00 (£**0.75**) based on how many ***CORRECT*** answers you provide regarding the accuracy of the articles. Correct answers are based on the expert evaluations of non-partisan fact-checkers.

More specifically, we have pre-tested these articles to see how much they are liked by [Democrats/Republicans]. We want to see how close your answers are to the answers we identified in the pre-test. If you correctly identify 15 or more out of 16 articles that are liked by [Democrats/Republicans], you will receive the full bonus payment of $1.00. If you correctly identify 13 or more of the 16 articles that are liked by [Democrats/Republicans], you will receive a partial bonus payment of $0.50.

Additionally, if you answer 15 or more out of the 16 questions about accuracy correctly, you will receive the full bonus payment of $1.00. If you answer 13 or more of the 16 questions correctly, you will receive a partial bonus payment of $0.50. Your bonus payment will be delivered to your Prolific ID. It may take a few weeks to calculate your scores and for you to receive your bonus payment.

We ask you about accuracy on a 6-point scale ranging from "extremely inaccurate" to "extremely accurate." For the purpose of this study, if the headline describes a true event, either "slightly accurate," "moderately accurate," or "extremely accurate" constitute correct responses. Similarly, if the headline describes a false event, either "extremely inaccurate," "moderately inaccurate," or "slightly inaccurate" constitute "correct" responses.

We will also ask you:

3) How likely would you be to share the headline on social media?

But, we will not give you a bonus payment based on your response to this question. Your answers to all other questions in the survey will not contribute to your bonus payment.

After seeing each headline, questions in this condition appeared as follows:

Note: If you correctly predict whether this headline will be liked by Democrats in the below question, you have a higher chance of receiving a bonus payment.

If you shared this article on social media, how likely is it that it would receive a positive reaction from Democrats (e.g., likes, shares, and positive comments)?

Very unlikely	Moderately unlikely	Slightly unlikely	Slightly likely	Moderately likely	Very likely

Note: If you answer the question below about accuracy correctly, you have a higher chance of receiving a bonus payment.

To the best of your knowledge, is the claim in the above headline accurate?

Not at all accurate	Moderately innaccurate	Slightly inaccurate	Slightly accurate	Moderately accurate	Extremely accurate

If you were to see the above article on social media, how likely would you be to share it?

Extremely unlikely	Moderately unlikely	Slightly unlikely	Slightly likely	Moderately likely	Extremely likely

Non-Financial Accuracy Incentive Manipulation Text

You will be presented with a series of real and fake news headlines. There are 16 headlines in total.

We are interested in your opinion about the following:

1) How accurate is the headline?

You will be given 60 seconds to answer this question about each headline.

Note: These are questions that have right and wrong answers. In order for your answers to be most helpful to us, it is really important that you answer these questions as accurately as you can.

At the end of the study, you will ***receive feedback*** on how many correct answers you provide regarding the accuracy of the articles. Correct answers are based on the expert evaluations of non-partisan fact-checkers.

We ask you about accuracy on a 6-point scale ranging from "extremely inaccurate" to "extremely accurate." For the purpose of this study, if the headline describes a true event, either "slightly accurate," "moderately accurate," or "extremely accurate" constitute correct responses. Similarly, if the headline describes a false event, either "extremely inaccurate," "moderately inaccurate," or "slightly inaccurate" constitute "correct" responses.

We will also ask you:

2) How likely would you be to share the headline on social media?

The images may take a second to load. Please wait for the images to load before answering the questions.

[page break]

Before you begin, we would like to share some related research about this topic.

Americans believe it is important to hold accurate beliefs about the news. For example, 89% of Americans believe it is “very important” for a news outlet to be accurate. 86% said that it is “very important” that they correct their mistakes. Additionally, there is clear bipartisan support for sharing accurate news–both Republicans and Democrats believe it is very important to only share accurate news.

Sharing fake news is actually extremely rare. One study found that 0.1% of people accounted for 80% of fake news shared! In other words, the vast majority of people do not share fake news.

Spreading fake news hurts your reputation. One study found that the majority of people would not even want to share fake news if they received money, because they believed that it would hurt their reputation. In fact, 50% of people say they avoided someone because they thought they would bring up fake news in conversation.

If you are interested in learning more, additional information about the research behind these topics will be available at the end of the study.

After seeing each headline, questions in this condition appeared as follows:

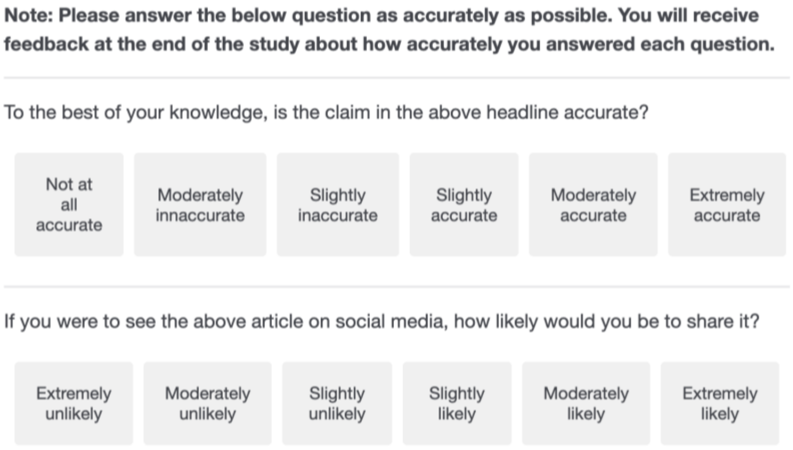

Note: Please answer the below question as accurately as possible. You will receive feedback at the end of the study about how accurately you answered each question.

To the best of your knowledge, is the claim in the above headline accurate?

Not at all accurate	Moderately innaccurate	Slightly inaccurate	Slightly accurate	Moderately accurate	Extremely accurate

If you were to see the above article on social media, how likely would you be to share it?

Extremely unlikely	Moderately unlikely	Slightly unlikely	Slightly likely	Moderately likely	Extremely likely

S3: Headline-Level Analysis

Type	Headline	Source	Mean (Accuracy)	Mean (Control)	Mean Difference	CI Low	CI High	t	df	p	Cohen's D
Democrat True	Trump allies are handing out cash to black voters	Politico	0.38	0.29	0.09	0.05	0.13	4.33	2065.73	0.000	0.19
Democrat True	Trump targets Reagan foundation after it asks campain, RNC to stop using former president's likeness	CBS News	0.68	0.54	0.13	0.09	0.17	6.32	2088.84	0.000	0.28
Democrat True	Facebook removes Trump ads with symbols once used by Nazis	AP News	0.63	0.54	0.08	0.04	0.13	3.94	2089.74	0.000	0.17
Democrat True	Melania Trump was praised for acknowledging racism. But she has also spread false 'birther' claims about Trump.	Washington Post	0.69	0.59	0.10	0.06	0.14	4.89	2088.95	0.000	0.21
Democrat False	White House Chef Quits because Trump Has Only Eaten Fast Food For 6 Months	HalfWay Post	0.20	0.19	0.00	-0.03	0.04	0.28	2084.83	0.777	0.01
Democrat False	Trump's Top Scientist Pick: "Scientists Are Just Dumb Regular People That Think Dinosaurs Existed and the Earth is Getting Warmer"	USPoln	0.25	0.22	0.04	0.00	0.07	1.90	2072.18	0.057	0.08
Democrat False	Hispanic Women Claims, "Donald Trump Paid Me For Sex in Cancun, This Is Our Love Child"	Now8News	0.17	0.11	0.06	0.03	0.09	3.88	1998.94	0.000	0.17
Democrat False	Donald Trump Signs Executive Order Allowing the Hunting of Bald Eagles	St. George Gazette	0.14	0.15	-0.01	-0.04	0.02	-0.55	2089.44	0.585	-0.02
Republican True	Plant a Million Trees: Republicans Offer Fossil-Friendly Climate Fix	Reuters	0.53	0.45	0.08	0.04	0.12	3.76	2086.16	0.000	0.16
Republican True	UPSP Flashback: Obama administration removed thousands of mailboxes	Fox News	0.48	0.35	0.13	0.09	0.18	6.24	2074.51	0.000	0.27
Republican True	Trump gets support of NYC police union, warns 'no one will be safe in Biden's America'	NBC News	0.85	0.79	0.06	0.03	0.10	3.78	2070.67	0.000	0.17
Republican True	Chinese dissedent brought to US by Obama administration praises Trump at RNC	CNN	0.51	0.42	0.09	0.04	0.13	3.98	2084.27	0.000	0.17
Republican False	UPDATE: Malia Obama Among 10 Arrested In Racist Antifa Attach -- US NEWS	PoliceUS.Info	0.09	0.10	-0.01	-0.04	0.01	-0.87	2089.74	0.384	-0.04
Republican False	Hillary Clinton Accepted $30,000 Donation From NXIVM Child Sex Cult	YourNewsWire	0.30	0.28	0.02	-0.02	0.06	1.07	2082.04	0.286	0.05

Republican False	The 'Obama Foundation' Just Broke Its First Federal Law	WeaponStricks	0.30	0.29	0.00	-0.04	0.04	0.11	2086.24	0.910	0.00
Republican False	Donald Trump Sent His Own Plane To Transport 200 Stranded Marines	Uconservative	0.42	0.44	-0.02	-0.06	0.03	-0.71	2087.33	0.481	-0.03

Note. Data is from the integrative data analysis. Independent samples t-tests with two-sided tests are reported. No multiple comparisons were applied to this analysis. As shown above, incentives significantly increased the perceived accuracy of each true article, but did not decrease the perceived accuracy of any false news article. However, incentives increased the perceived accuracy of one false news article.

S4: Bayes Factors for Integrative Data Analysis

We used the "BayesFactor" package in R to conduct additional Bayesian t-tests. This package by default places a noninformative Jeffreys prior on the variance of the normal population, and a Cauchy prior on the standardized effect size.

The Bayes Factor (BF) for the amount of politically-incongruent true news rated as true in the experimental as compared to the control condition was 3.17×10^{22}±0%, showing extreme evidence for the alternative hypothesis. The BF for the amount of politically-congruent true news rated as true was 132.26 ±0%, once again showing extreme evidence for the alternative hypothesis. The BF for the amount of politically-incongruent false news rated as true was 11.43±0%, showing strong evidence for the alternative hypothesis. The BF for the amount of politically-congruent false news rated as true was 0.07±0.34%, indicating moderate evidence for the null hypothesis.

The BF for truth discernment is $6.46\text{x}10^{7}$, showing extreme evidence for the alternative hypothesis. The BF for partisan bias is $1.28\text{x}10^{6}$, also showing extreme evidence for the alternative hypothesis. The BF for sharing discernment is 0.53±0.01%, indicating anecdotal evidence for the null hypothesis.

The BF for the interaction between political party and the condition on the amount of politically-incongruent true news rated as true is 0.68±0.01%. The BF for this interaction on partisan bias is 0.76±0.01%. For truth discernment as the outcome variable, this interaction is 0.27±0.01%. Lastly, for sharing discernment as the outcome variable, this interaction is 0.54±0.04% For all the moderation effects, the Bayes factors fall in the range of anecdotal to moderate evidence for the null hypothesis.

S5: Mean Effects Sizes for All Participants and Separately for Republicans and Democrats

Variable	*Cohen's D*	95% CI	*p*
	All Participants (n = 2,092)		
Truth Discernment	0.29	[0.20, 0.37]	< 0.001
Partisan Bias	0.26	[0.17, 0.34]	< 0.001
Incongruent True News	0.47	[0.38, 0.55]	< .001
Sharing Discernment	0.10	[0.00, 0.12]	0.029
	Liberals (n = 1,166)		
Truth Discernment	0.23	[0.11, 0.35]	< 0.001
Partisan Bias	0.20	[0.09, 0.32]	< 0.001
Incongruent True News	0.40	[0.29, 0.52]	< 0.001
Sharing Discernment	0.02	[-0.06, 0.09]	0.718
	Conservatives (n = 926)		
Truth Discernment	0.40	[0.26, 0.53]	< 0.001
Partisan Bias	0.34	[0.21, 0.47]	< 0.001
Incongruent True News	0.57	[0.44, 0.71]	< 0.001
Sharing Discernment	0.12	[0.05, 0.19]	0.002

Note. Effects of the incentives shown for all participants using data from the integrative data analysis and shown separately for liberals and conservatives. Independent samples t-tests with two-sided tests are reported. No multiple comparisons were applied to this analysis.

S6: Full Regression Models for Integrative Data Analysis

	Truth Discernment	Partisan Bias	Incongruent True News	Sharing Discernment
(Intercept)	-0.09 (0.008)	0.04 (0.267)	-0.14 (< 0.001)	0.04 (0.274)
	[-0.16, -0.02]	[-0.03, 0.11]	[-0.21, -0.07]	[-0.03, 0.12]
conditionRecode	0.27 (<0.001)	-0.24 (<0.001)	0.45 (< 0.001)	0.10 (0.021)
	[0.19, 0.35]	[-0.32, -0.16]	[0.37, 0.53]	[0.02, 0.19]
PoliticalOrientation	-0.28 (<0.001)	0.32 (<0.001)	-0.21 (< 0.001)	-0.10 (< 0.001)
	[-0.33, -0.24]	[0.27, 0.36]	[-0.25, -0.16]	[-0.14, -0.05]
CRSum	0.14 (<0.001)	-0.07 (0.001)	0.08 (< 0.001)	-0.03 (0.211)
	[0.10, 0.18]	[-0.11, -0.03]	[0.04, 0.12]	[-0.07, 0.02]
PKSum	0.15 (<0.001)	-0.02 (0.271)	0.07 (0.003)	0.08 (< 0.001)
	[0.11, 0.19]	[-0.07, 0.02]	[0.02, 0.11]	[0.03, 0.13]
outgrouphate	0.07 (0.002)	0.22 (0.000)	-0.08 (0.001)	-0.03 (0.221)
	[0.02, 0.11]	[0.18, 0.26]	[-0.12, -0.03]	[-0.07, 0.02]
Education	-0.00 (0.877)	-0.02 (0.268)	0.05 (0.021)	-0.02 (0.337)
	[-0.05, 0.04]	[-0.07, 0.02]	[0.01, 0.10]	[-0.07, 0.02]
Age	-0.07 (<0.001)	0.10 (<0.001)	-0.10 (0.001)	-0.05 (0.025)
	[-0.11, -0.03]	[0.06, 0.14]	[-0.14, -0.05]	[-0.10, -0.01]
Income	0.02 (0.457)	-0.01 (0.691)	0.01 (0.575)	-0.02 (0.446)
	[-0.03, 0.06]	[-0.05, 0.03]	[-0.03, 0.06]	[-0.06, 0.03]
GenderRecode	-0.08 (0.062)	0.15 (<0.001)	-0.15 (0.001)	-0.18 (<0.001)
	[-0.16, 0.00]	[0.07, 0.23]	[-0.24, -0.07]	[-0.27, -0.09]
N	2041	2041	2041	2041
R2	0.19	0.18	0.14	0.03

Note. Multiple regression models for integrative data analysis. Standardized effect sizes are shown for ease of interpretation. P-values are shown next to effect sizes, and 95% confidence intervals are shown below.

S7: Full Relative Importance Analysis for Integrative Data Analysis

Truth Discernment

term	lmg	conf.low	conf.high
conditionRecode	0.266	0.005	0.864
PoliticalOrientation	0.834	0.203	1.855
CRSum	0.030	0.017	0.275
PKSum	0.472	0.085	1.157
outgrouphate	0.034	0.011	0.368
Education	0.037	0.004	0.378
Age	0.305	0.020	0.900
Income	0.035	0.004	0.342
GenderRecode	0.730	0.176	1.650

Partisan Bias

term	lmg	conf.low	conf.high
conditionRecode	1.496	0.680	2.558
PoliticalOrientation	8.916	6.711	11.341
CRSum	0.823	0.257	1.584
PKSum	0.062	0.047	0.287
outgrouphate	3.532	2.254	5.022
Education	0.256	0.044	0.767
Age	1.790	0.825	2.979
Income	0.040	0.018	0.279
GenderRecode	0.712	0.229	1.526

Incongruent True News

term	lmg	conf.low	conf.high
conditionRecode	5.108	3.329	7.132
PoliticalOrientation	4.188	2.851	6.040
CRSum	1.080	0.435	2.089
PKSum	0.427	0.095	1.067
outgrouphate	0.313	0.068	1.002
Education	0.502	0.097	1.283
Age	1.165	0.460	2.190
Income	0.074	0.018	0.440
GenderRecode	0.772	0.239	1.714

Incongruent True News

term	lmg	conf.low	conf.high
conditionRecode	0.266	0.010	0.920
PoliticalOrientation	0.834	0.236	1.780
CRSum	0.030	0.017	0.301
PKSum	0.472	0.087	1.169
outgrouphate	0.034	0.012	0.364
Education	0.037	0.004	0.355
Age	0.305	0.037	0.941
Income	0.035	0.004	0.395
GenderRecode	0.730	0.207	1.625

S8: Example Stimuli

Example Republican-Leaning Real News:

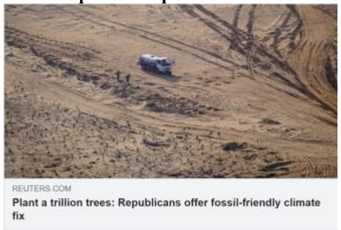

Example Democrat-Leaning Real News:

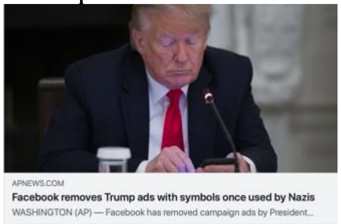

Example Democrat-Leaning Fake News

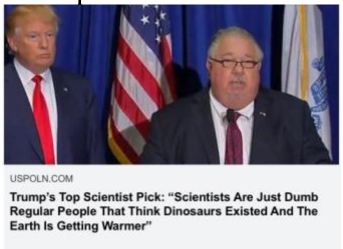

Example Republican-Leaning Fake News:

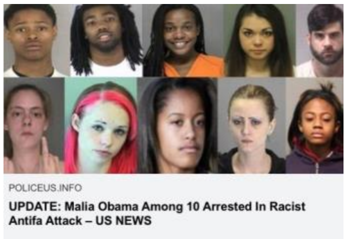

Example headline without source:

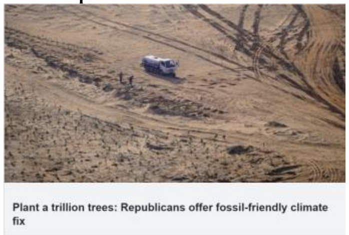

The full set of stimuli are available in our OSF:
https://osf.io/75sqf/?view_only=623d654c17b94d958a0857c06b181073
All stimuli are from the OSF page (https://osf.io/xyq4t/) from this paper:
Pennycook, G., Binneddyk, J., Netwon, C., & Rand, D.G. (2021), A practical guide to doing behavioral research on fake news and misinformation. Collabra: Psychology, 7(1), 25293.
https://online.ucpress.edu/collabra/article/7/1/25293/117809/A-Practical-Guide-to-Doing-Behavioral-Research-on

S9: Question Wording

Cognitive Reflection Test.
The ages of Mark and Adam add up to 28 years total. Mark is 20 years older than Adam. How many years old is Adam?
If it takes 10 seconds for 10 printers to print out 10 pages of paper, how many seconds will it take 50 printers to print out 50 pages of paper?
On a loaf of bread, there is a patch of mold. Every day, the patch doubles in size. If it takes 40 days for the patch to cover the entire loaf of bread, how many days would it take for the patch to cover half of the loaf of bread?

Affective Polarization.
How favorable do you feel towards Democrats?
How favorable do you feel towards Republicans?
Note: Affective polarization was measured as positive feelings toward the in-party minus negative feelings toward the out-party.

Political Knowledge.
Whose responsibility is it to determine if a law is constitutional or not – is it the President, the Congress, or the Supreme Court?
President
Congress
Supreme Court
How much of a majority is required for the U.S. Senate and House to override a presidential veto?
1/2 majority
1/3 majority
3/4 majority
What party currently has the most members in the House of Representatives in Washington?
Democrats
Republicans
Neither
Would you say that one of the major parties is more conservative than the other at the national level? If so, which party is more conservative?
Democrats
Republicans
Neither
How many justices are on the U.S. Supreme Court?
9
12
18

Political Conservatism.
Which of the following best describes your political preference?
Strongly Democratic
Democratic

Lean Democratic
Lean Republican
Republican
Strongly Republican

Education.
What is the highest level of school you have completed or the highest degree you have received?
Less than high school degree
High school graduate (high school diploma or equivalent including GED)
Some college but no degree
Associate degree in college (2-year)
Bachelor's degree in college (4-year)
Master's degree
Doctoral degree
Professional degree (JD, MD)

Income.
Information about income is very important to understand. Would you please give your best guess?
Please indicate the answer that includes your entire household income in (previous year) before taxes.
Less than $10,000
$10,000 to $19,999
$20,000 to $29,999
$30,000 to $39,999
$40,000 to $49,999
$50,000 to $59,999
$60,000 to $69,999
$70,000 to $79,999
$80,000 to $89,999
$90,000 to $99,999
$100,000 to $149,999
$150,000 or more

Age.
What is your age?

Gender (Recoded for Regression Analysis as Female/Not Female)
What is your gender?
Male
Female
Transgender Female
Transgender Male
Trans/Non-Binary
Not Listed:

Additional Questions

Did you respond randomly at any point during the study?

Note: Please be honest! You will get your payment regardless of your response.

Yes

No

…

Please answer honestly:

Would you ever share an article on social media that you know is false?

Yes

No

Note: These questions were only shown to those in the accuracy incentives condition:

Please answer honestly: this will not affect your payment, and it is important to the researchers to understand how you responded.

Did you ever say an article was accurate simply because you thought it would get you a higher payment and not because you genuinely believed it was accurate?

Or did all answers about accuracy reflect your true beliefs?

Yes

No

…

Please answer honestly: do you think your answers were influenced by the extra financial incentive to be accurate?

Yes

No

Note: These questions were only shown to those in the social incentives condition:

Please answer honestly: this will not affect your payment, and it is important to understand how you responded.

Did you ever say an article was accurate simply because you liked it (or say an article was inaccurate because you disliked it)?

Or did all answers about accuracy reflect your true beliefs?

Yes

No

…

Please answer honestly: do you think your answers to other questions were influenced by your task of identifying articles that would appeal to your political party?

Yes

No

S10: Results for Continuously Coded Outcome Variables

	Mean (Accuracy)	Mean (Control)	Difference	CI_low	CI_high	t	df	p	d
Truth Discernment	0.81	0.70	0.11	0.05	0.17	3.62	2062.22	< 0.001	0.16
Partisan Bias	0.58	0.76	-0.18	-0.27	-0.08	-3.71	2058.16	< 0.001	-0.16
Incongruent True News	3.95	3.76	0.19	0.10	0.28	4.03	2083.40	< 0.001	0.18

Note. Above are the results for truth discernment, partisan bias, and incongruent true news when coded on a continuous, rather than dichotomous scale. Results shown above are from the integrative data analysis. Two-sided independent samples t-tests are reported, and no adjustments were made for multiple comparison. Results do not change the conclusions, but the effect sizes are smaller. This smaller effect can possibly be attributed to the fact that incentives rewarded responses as accurate regardless of whether people answered “slightly accurate,” “moderately accurate,” or “extremely accurate.” In other words, people may have been ignoring magnitude when answering questions.

S11: Study 3 Results Including Additional News Items

Below, we show the results for Study 3 when including the 8 additional news stimuli. These additional analyses do not change our conclusions.

Truth Discernment. A 2X2 (Accuracy X Source) ANOVA found that there was a significant effect of the accuracy incentives on truth discernment such that the accuracy incentives improved discernment, $F(1, 917) = 4.08$, $p = 0.04$, $\eta^2_G = 0.004$. Additionally, there was a significant impact of source cues on truth discernment such that source cues improved accuracy, $F(1, 917) = 8.13$, $p = 0.004$, $\eta^2_G = 0.009$. However, there was no significant interaction between the accuracy incentive condition and source cues, $p = 0.649$.

Partisan Bias. A 2X2 (Accuracy X Source) ANOVA found that there was a significant effect of the accuracy incentives on partisan bias such that the incentives reduced bias, $F(1, 917) = 16.79$, $p < 0.001$, $\eta^2_G = 0.02$. The source cues did not impact partisan bias ($p = 0.603$), and there was no interaction between source cues and partisan bias ($p = 0.438$).

Sharing Discernment. A 2X2 (Accuracy X Source) ANOVA found that there was no significant effect of accuracy incentives ($p = 0.733$), but there was a significant effect of the source cues, $F(1, 917) = 5.50$, $p = 0.019$, $\eta^2_G = 0.01$, and no interaction between accuracy incentives and source cues (p = 0.533).

Effects on Sharing Intentions Broken Down by Headline Type. We then ran a 2 (accuracy incentive vs. no incentive) X 2 (social incentive vs. no incentive) X 2 (politically congruent vs. politically incongruent) X 2 (true headlines vs. false headlines) mixed-design ANOVA. We found no main effect of source cues ($p = 0.524$), but did find a main effect of the incentive, $F(1,917) = 0.396$, $p = 0.047$, $\eta^2_G = 0.00$, political congruence, $F(1, 917) = 5.79$, $p = 0.016$, $\eta^2_G = 0.00$, and veracity, $F(1, 917) = 1330.58$, $p < 0.001$, $\eta^2_G = 0.24$. There was also a significant interaction between the incentives and political congruence, $F(1, 917) = 4.12$, $p = 0.043$, $\eta^2_G = 0.00$, the source cues and veracity, $F(1, 917) = 8.18$, $p = 0.004$, $\eta^2_G = 0.00$, incentives and veracity, $F(1, 917) = 4.06$, $p = 0.044$, $\eta^2_G = 0.00$, and political congruence and veracity, $F(1, 917) = 361.44$, $p < 0.001$, $\eta^2_G = 0.09$. There were also significant three-way interactions between source cues, incentives, and political congruence, $F(1, 917) = 5.00$, $p = 0.026$, $\eta^2_G = 0.00$, and incentives, political congruence, and veracity, $F(1, 917) = 16.74$, $p = 0.00$, $\eta^2_G = 0.01$.

Importantly, post-hoc Tukey HSD tests revealed that there was a difference between belief in *incongruent true* headlines rated as accurate in the accuracy incentive (with sources) condition (M = 56.33, 95% CI = [52.92, 59.75]) versus the control (with sources) condition ($M = 46.24$, 95% CI = [42.79, 49.70]), $p < 0.001$, $d = 0.39$. However, there was no difference between the accuracy incentives condition (without sources cues) as compared to the control condition (without source cues), $p = 0.311$.

In sum, we found the same conclusions for Study 3 when including these additional stimuli in our analysis.

S12: Signal Detection Analysis

Following Batailler et al. (2021), we also performed signal detection analysis. First, we calculated *d'*, or discrimination sensitivity, which aims to capture participants' ability to correctly discriminate between true news and fake news. A higher *d'* indicates a better ability to correctly discriminate between true news headlines and fake news headlines. This measure is conceptually similar to truth discernment, and is calculated the following way:

$$d' = z(hits) - z(false\ alarms)$$

Following Batailler et al. (2021), we applied a log-linear rule correction, adding 0.5 to all cells before calculating the above formula.

A 2 (Accuracy Incentive vs. Condition) X 2 (Politically Congruent vs. Politically Incongruent) ANOVA found that the incentives condition increased discrimination sensitivity, $F(1, 2090) = 41.65$, $p < 0.001$, $\eta^2_G = 0.01$. There was no main effect of political congruence, $F(1, 2090) = 2.14$, $p = 0.144$, $\eta^2_G = 0.00$. However, the accuracy incentives significantly interacted with political congruence, $F(1, 2090) = 7.53$, $p = 0.006$, $\eta^2_G = 0.00$. Following up on this interaction, Tukey post-hoc tests found that *d'* was significantly higher for both politically-congruent headlines (*M* = 0.87, 95% CI = [0.82, 0.93]) in the accuracy incentives condition as compared to the control condition (*M* = 0.72, 95% CI = [0.66, 0.78]), *d* = 0.16, $p < 0.001$. Additionally, d' was significantly higher for politically-incongruent headlines in the accuracy incentives condition (*M* = 0.90, 95% CI = [0.85, 0.95]) as compared to the control condition (*M* = 0.63, 95% CI = [0.59, 0.68]), *d* = 0.33, $p < 0.001$. In sum, incentive improved discrimination sensitivity for both politically-congruent and politically-incongruent headlines, but the effect was significantly stronger for politically-incongruent headlines.

Then, also following Batailler et al. (2021), we calculated *c'*, or response bias, which aims to capture the threshold of perceived veracity that a given news headline must reach before someone accepts a headline is true. A higher (or more conservative) *c'* value indicates that a participant is less likely to judge any given headline as true, and a lower (or more liberal) *c'* value indicates that a participant is more likely to judge any given headline as true.

$$c = -1 \times \frac{z(hits) - z(false\ alarms)}{2}$$

A 2 (Accuracy Incentive vs. Control) X 2 (Politically Congruent vs. Politically Incongruent) ANOVA found that the incentives condition increased response bias, $F(1, 2090) = 48.76$, $p < 0.001$, $\eta^2_G = 0.01$. Similar to Batailler et al., (2021), we found that political congruence had a very large effect on response bias, $F(1, 2090) = 1089.94$, $p < 0.001$, $\eta^2_G = 0.15$. Additionally, there was an interaction between accuracy incentives and political congruence, $F(1, 2090) = 34.96$, $p < 0.001$, $\eta^2_G = 0.00$. Following up on this interaction, Tukey post-hoc tests found that c' was not significantly higher for politically congruent news as compared to the control condition, $p = 0.152$. However, it was significantly higher for politically-incongruent news (*M* = 0.41, 95% CI = [0.38, 0.44]) as compared to the control condition (*M* = 0.62, 95% CI = [0.59, 0.66]), $p < 0.001$, *d* = 0.41. In sum, incentives decreased participants' threshold for judging politically-incongruent headlines as true.

Supplementary References

Batailler, C., Brannon, S. M., Teas, P. E., & Gawronski, B. (2021). A signal detection approach to understanding the identification of fake news. *Perspectives on Psychological Science*, *22*.

Gawronski, B. (2021). Partisan bias in the identification of fake news. *Trends in Cognitive Sciences*, *25*(9), 723–724.

Pennycook, G., & Rand, D. G. (2018). Lazy, not biased: Susceptibility to partisan fake news is better explained by lack of reasoning than by motivated reasoning. *Cognition*. https://doi.org/10.1016/j.cognition.2018.06.011

Pennycook, G., & Rand, D. G. (2021a). Lack of partisan bias in the identification of fake (versus real) news. *Trends in Cognitive Sciences*, *25*(9), 725–726.

Pennycook, G., & Rand, D. G. (2021b). The psychology of fake news. *Trends in Cognitive Sciences*.

Peterson, E., & Iyengar, S. (2021). Partisan Gaps in Political Information and Information-Seeking Behavior: Motivated Reasoning or Cheerleading? *American Journal of Political Science*, *65*(1), 133–147.

Schaffner, B. F., & Luks, S. (2018). Misinformation or expressive responding? What an inauguration crowd can tell us about the source of political misinformation in surveys. *Public Opinion Quarterly*, *82*(1), 135–147.
