# Supplementary material for: Accuracy and social motivations shape judgements of (mis)information
Source: Nat Hum Behav. 2023 Mar 6;7(6):892–903. doi: 10.1038/s41562-023-01540-w (PMC10289897; doi:10.1038/s41562-023-01540-w)
Supplement: Supplementary file 2 — Reporting Summary [file 41562_2023_1540_MOESM2_ESM.pdf]

nature portfolio

Corresponding author(s): Steve Rathje

Last updated by author(s): Jan 2, 2022

Reporting Summary

Nature Portfolio wishes to improve the reproducibility of the work that we publish. This form provides structure for consistency and transparency in reporting. For further information on Nature Portfolio policies, see our Editorial Policies and the Editorial Policy Checklist.

Statistics

For all statistical analyses, confirm that the following items are present in the figure legend, table legend, main text, or Methods section.

n/a	Confirmed	
☐	☒	The exact sample size (n) for each experimental group/condition, given as a discrete number and unit of measurement
☐	☒	A statement on whether measurements were taken from distinct samples or whether the same sample was measured repeatedly
☐	☒	The statistical test(s) used AND whether they are one- or two-sided<br>*Only common tests should be described solely by name; describe more complex techniques in the Methods section.*
☐	☒	A description of all covariates tested
☐	☒	A description of any assumptions or corrections, such as tests of normality and adjustment for multiple comparisons
☐	☒	A full description of the statistical parameters including central tendency (e.g. means) or other basic estimates (e.g. regression coefficient) AND variation (e.g. standard deviation) or associated estimates of uncertainty (e.g. confidence intervals)
☐	☒	For null hypothesis testing, the test statistic (e.g. F, t, r) with confidence intervals, effect sizes, degrees of freedom and P value noted<br>*Give P values as exact values whenever suitable.*
☐	☒	For Bayesian analysis, information on the choice of priors and Markov chain Monte Carlo settings
☒	☐	For hierarchical and complex designs, identification of the appropriate level for tests and full reporting of outcomes
☐	☒	Estimates of effect sizes (e.g. Cohen's d, Pearson's r), indicating how they were calculated

Our web collection on statistics for biologists contains articles on many of the points above.

Software and code

Policy information about availability of computer code

Data collection	All data was collected via the survey platform Qualtrics in 2020-2021 (Version 2020 and 2021).
Data analysis	All data were analyzed using using R version 4.01. A number of R packages were used for analysis, such as rstatix (stats and effect sizes, version 0.6.0), relampo (for relative importance analysis, verison 2.2.5), ggplot2 (for plotting, version 3.4.0), BayesFactor (for Bayesian analysis, version 0.9.12.3), and jtools (for regression, version 2.1.4). Analysis code is available at our OSF: https://osf.io/75sqf.

For manuscripts utilizing custom algorithms or software that are central to the research but not yet described in published literature, software must be made available to editors and reviewers. We strongly encourage code deposition in a community repository (e.g. GitHub). See the Nature Portfolio guidelines for submitting code & software for further information.

Data

Policy information about availability of data

All manuscripts must include a data availability statement. This statement should provide the following information, where applicable:

- Accession codes, unique identifiers, or web links for publicly available datasets
- A description of any restrictions on data availability
- For clinical datasets or third party data, please ensure that the statement adheres to our policy

Anonymized data, Qualtrics files, and stimuli are available on the Open Science Framework (OSF): https://osf.io/75sqf.

Human research participants

Policy information about studies involving human research participants and Sex and Gender in Research.

Reporting on sex and gender	All participants reported their gender, but not their biological sex, as it was not considered essential to this analysis. was not considered essential Participants were given the opportunity to identify as male, female, transgender male, transgender female, non-binary, or other. In other multiple regression model, we included gender as a covariate, along with other demographic covariates, such as age and political affiliation. We had no prior hypotheses about the role of sex and gender in our research questions.
Population characteristics	See above
Recruitment	For Study 1, 2, and 4, the samples were convenience samples collected via the survey platform Prolific Academic. For Study 3, the sample was a nationally representative sample collected via the survey platform Prolific Academic. Since the data was collected via Prolific, it could have self-selection biases informed by the users of Prolific. For instance, it may over-representative of internet-savy researchers who know about Prolific and are interested in taking surveys. Our survey with a nationally representative sample (Study 3) helps protect against these confounds.
Ethics oversight	The research methods were approved by the University of Cambridge Psychology Ethics Committee (Protocol #PRE.2020.110).

Note that full information on the approval of the study protocol must also be provided in the manuscript.

Field-specific reporting

Please select the one below that is the best fit for your research. If you are not sure, read the appropriate sections before making your selection.

☐ Life sciences ☒ Behavioural & social sciences ☐ Ecological, evolutionary & environmental sciences

For a reference copy of the document with all sections, see nature.com/documents/nr-reporting-summary-flat.pdf

Behavioural & social sciences study design

All studies must disclose on these points even when the disclosure is negative.

Study description	Four experiments investigating whether motivating people to be accurate (for example, via financial incentives) changes perceptions of true and false news.
Research sample	Studies 1, 2, and 4 have politically-balanced samples recruited via Prolific Academic (e.g., half-Democrat, half-Republican). For Study 3, a nationally-representative (quota-matched to the US distribution of age, gender, political party, and race/ethnicity) sample was collected via Prolific Academic.
Sampling strategy	Data was collected via the survey platform Qualtrics, and the sample was collected via Prolific. For Study 1, 2, and 4, the samples were convenience samples collected via the survey platform Prolific Academic. For Study 3, the sample was a nationally representative sample collected via the survey platform Prolific Academic.
Data collection	Data was collected via the survey platform Qualtrics. Because all randomization happened automatically through Qualtrics, the researcher was effectively blind to the study condition when collecting data.
Timing	Study 1 data was collected on Nov. 30, 2020, Study 2 data was collected on Jan. 22, 2021, and Study 3 data was collected on June 13, 2021. Stopping times were determined by when Prolific Academic hit our target sample size.
Data exclusions	l exclusion criteria were preregistered. In Study 1, 32 participants were excluded for failing an attention check at the end of the survey (or not getting to that point in the survey). An additional 17 participants were also excluded from Study 1 for reporting responding randomly at any time during the experiment. In Study 2, 76 participants were excluded for failing our attention check/not completing the survey, and an additional 39 participants were excluded for reporting responding randomly at some point during the survey. In Study 3, 95 participants were excluded for failing the attention check/not completing the survey, and an additional 39 participants were excluded for reporting responding randomly at some point during the survey. In Study 4, we excluded 16 participants who failed our attention check (or did not finish enough of the survey to reach the attention check) and an additional 8 participants who said they responded randomly at some point during the experiment.
Non-participation	Study 1, 10 participants did not complete the survey. In Study 2, 14 participants did not complete the survey. In Study 3, 40 participants did not complete the survey. In Study 4, 6 participants did not complete the survey. These participants were not included for analysis as they were captured by our exclusion criteria.
Randomization	In all studies, participants were randomized to experimental condition via Qualtric's randomization feature.

Reporting for specific materials, systems and methods

We require information from authors about some types of materials, experimental systems and methods used in many studies. Here, indicate whether each material, system or method listed is relevant to your study. If you are not sure if a list item applies to your research, read the appropriate section before selecting a response.

Materials & experimental systems

n/a	Involved in the study
☒	☐ Antibodies
☒	☐ Eukaryotic cell lines
☒	☐ Palaeontology and archaeology
☒	☐ Animals and other organisms
☒	☐ Clinical data
☒	☐ Dual use research of concern

Methods

n/a	Involved in the study
☒	☐ ChIP-seq
☒	☐ Flow cytometry
☒	☐ MRI-based neuroimaging
